# Partitioning Apomixis Components to Understand and Utilize Gametophytic Apomixis

**DOI:** 10.3389/fpls.2019.00256

**Published:** 2019-03-08

**Authors:** Pankaj Kaushal, Krishna K. Dwivedi, Auji Radhakrishna, Manoj K. Srivastava, Vinay Kumar, Ajoy Kumar Roy, Devendra R. Malaviya

**Affiliations:** ^1^ICAR-National Institute of Biotic Stress Management, Raipur, India; ^2^ICAR-Indian Grassland and Fodder Research Institute, Jhansi, India; ^3^ICAR-Indian Institute of Sugarcane Research, Lucknow, India

**Keywords:** apomixis, apomeiosis, endosperm, *Panicum maximum*, parthenogenesis, partitioning

## Abstract

Apomixis is a method of reproduction to generate clonal seeds and offers tremendous potential to fix heterozygosity and hybrid vigor. The process of apomictic seed development is complex and comprises three distinct components, viz., apomeiosis (leading to formation of unreduced egg cell), parthenogenesis (development of embryo without fertilization) and functional endosperm development. Recently, in many crops, these three components are reported to be uncoupled leading to their partitioning. This review provides insight into the recent status of our understanding surrounding partitioning apomixis components in gametophytic apomictic plants and research avenues that it offers to help understand the biology of apomixis. Possible consequences leading to diversity in seed developmental pathways, resources to understand apomixis, inheritance and identification of candidate gene(s) for partitioned components, as well as contribution towards creation of variability are all discussed. The potential of *Panicum maximum*, an aposporous crop, is also discussed as a model crop to study partitioning principle and effects. Modifications in cytogenetic status, as well as endosperm imprinting effects arising due to partitioning effects, opens up new opportunities to understand and utilize apomixis components, especially towards synthesizing apomixis in crops.

## Introduction

### Overview of Apomixis Phenomenon: Genetics and Regulation

Apomixis is a natural method of clonal reproduction through seeds, whereby the progeny is represented exactly by the maternal genotype (Asker and Jerling, 1992). This phenomenon has tremendous potential in agriculture by virtue of its capacity to fix heterozygosity and hybrid vigor (Sailer et al., 2016), it may equate an “*Asexual revolution*” (Calzada et al., 1996) and is proposed as a “*next generation breeding technology*” (Hand and Koltunow, 2014).

Apomixis is widespread in the plant kingdom and naturally occuring in 326 genera representing 78 families in Angiosperms, the majority belonging to Poaceae, Asteraceae and Rosaceae (Hojsgaard et al., 2014b). Apomixis may be of gametophytic or sporophytic origin based on the tissue involved in the formation of the female gametophyte. Gametophytic apomixis is represented by either diplospory or apospory, based on the origin of embryo-sacs (ES) in the ovule from megagametophyte or a nucellar cell, respectively (Nogler, 1984a; Asker and Jerling, 1992). When the mode of seed formation is exclusively through apomixis or sexual pathways, the plant is designated as obligate (apomictic or sexual, respectively). However, when both modes are represented in the same plant (co-exist in same ovule, or in different ovules of the same plant), it is regarded as facultative in reproduction.

The basic mechanism leading to the seed clonality relies on bypassing the two phases of variability generation, meiotic recombination and fertilization, during the seed formation, eventually resulting in seeds with copied maternal genotype. Meiosis is avoided (or eliminated/modified) via formation of apomeiotic embryo sacs (ES) while fertilization is bypassed via parthenogenetic development of the egg cell (Nogler, 1984a). Different modes of formation of apomeiotic ES to generate an unreduced egg cell have been widely discussed (Crane, 2001; Koltunow and Grossniklaus, 2003). Cyto-embryological and molecular processes were studied in model aposporous (e.g., *Brachiaria*, *Pennisetum*, *Cenchrus*, *Hieracium*, *Paspalum*, *Poa*, etc.) and diplosporous taxa (e.g., *Erigeron*, *Taraxacum*, *Tripsacum*, etc.), and important insights into the phenomenon have been presented (Grimanelli et al., 2001; Ozias-Akins and van Dijk, 2007; Pupilli and Barcaccia, 2012; Schmid et al., 2015; Schmidt et al., 2015; Hojsgaard, 2018).

The genetics of apomixis, as investigated in these model species, appeared broadly to be under the control of one or a few major dominant genes demonstrating Mendelian segregation (reviewed in Savidan, 2000; Ozias-Akins and van Dijk, 2007; Barcaccia and Albertini, 2013). In parallel, a Hybridization-derived Floral Asynchrony (HFA) hypothesis was also proposed advocating the apomictic phenotype as a result of asynchronous expression of duplicate genes controlling female gametophyte development (Carman, 1997).

Information on inheritance models, genetic recombination potentials, molecular markers and molecular mapping studies in gametophytic apomicts have been compiled in recent reviews (Ozias-Akins and van Dijk, 2007; Pupilli and Barcaccia, 2012; Barcaccia and Albertini, 2013; Hand and Koltunow, 2014; Brukhin, 2017; León-Martínez and Vielle-Calzada, 2019). In general, dominance, polyploidy, hybrid origin and suppressed recombination are common features related to apomixis in the majority of the apomictic species (Nogler, 1984a), e.g., Apospory Specific Genomic Region (*ASGR*) in *Pennisetum* spp. and *Cenchrus ciliaris* (Akiyama et al., 2005; Conner et al., 2008), Apomixis Controlling Locus (*ACL*) in *Paspalum simplex* (Calderini et al., 2006), Loss of Apomeiosis (*LOA*) in *Hieracium* subgenus *Pilosella* (Okada et al., 2011) and Apospory (*Apo*) locus in *Panicum maximum* (Ebina et al., 2005; Takahara et al., 2014).

Comparative gene expression studies including transcriptome analysis were conducted in many of these species and differentially expressed genes during different stages of apomictic and sexual seed formation were identified (reviewed in Brukhin, 2017; Conner and Ozias-Akins, 2017). Some candidate genes have been shortlisted as potential key genetic factors (Bicknell and Koltunow, 2004; Albertini et al., 2005; Laspina et al., 2008; Sharbel et al., 2010; Silveira et al., 2012; Okada et al., 2013).

Recent studies on gene expression network supported sexual and apomictic reproduction to be closely related developmental pathways. Apomixis is suggested to be a heterochronic phenotype which relies on deregulation of the timing of reproductive events (especially entry in apomeiosis/meiosis during ES development and parthenogenetic/zygotic embryogenesis), rather than on the alteration of a specific component of the reproductive pathway (Grimanelli et al., 2003; Tucker et al., 2003; Sharbel et al., 2010; Koltunow et al., 2011; Hojsgaard et al., 2012; Hojsgaard, 2018). The eventual expression of mode of reproduction is also believed to be regulated by modifiers, supernumerary chromatin and epigenetic modifications that may operate on account of hybridity and/or polyploidization (reviewed in Roche et al., 2001; Hand and Koltunow, 2014; Bocchini et al., 2018).

### Apomixis and Polyploidy

One of the key features on apomixis expression is its close relationship with polyploidy. In general, most of the naturally occurring apomictic species are polyploids, whereby lower forms (generally diploids) are sexually reproducing (Nogler, 1984a; Carman, 1997). However, recent recovery of natural and experimental diploids expressing apomixis indicate that though affected by the change in ploidy, apomixis expression is not restricted to polyploids (Visser and Spies, 1994; Siena et al., 2008; Lovell et al., 2013; Noyes and Wagner, 2014; Hojsgaard et al., 2014a; Schinkel et al., 2016, 2017; Klatt et al., 2018a). Effect of ploidy on apomixis expression has been studied in different apomictic systems using ploidy level variations at inter- or intra-specific levels (Savidan, 2000). Artificial polyploidization was observed to enhance (Quarin and Hanna, 1980; Quarin et al., 2001; Nassar, 2006) or reduce (Asker, 1967, 1980) its expression. Within a ploidy level, genotypic effects were more profound than ploidy effects in expressing mode of reproduction in many species such as *Poa pratensis*, *Boechera* spp., *Ranunculus kuepferi* and *Panicum maximum* (Matzk et al., 2005; Voigt-Zielinski et al., 2012; Schinkel et al., 2016; Kaushal et al., 2018). Such reports have contrasted the belief of ploidy-rise being the most important driver of apomixis evolution (Carman, 1997). In-fact, importance of hybridity over polyploidy, in governing apomixis, has been recently demonstrated (Delgado et al., 2016; Barke et al., 2018).

## The Apomixis Components and Their Uncoupling/Partitioning

### Components of Apomixis

Seeds of sexual origin generate from a meiotically derived ES, generally Polygonum-type (8-nucleated), containing a reduced egg cell (1*n*), which develops into an embryo (2*n*) after fertilization with a reduced male gamete (1*n*). Endosperm in such seeds is a triploid (3*n*) tissue which develops from fertilization of a male gamete (1*n*) with two fused polar nuclei (1*n* + 1*n* = 2*n*). This pathway of seed formation may be represented as *meiotic-ES:zygotic-embryogenesis:pseudogamous-endosperm*. In contrast, a generalized model on seed development though gametophytic apomixis (apospory or diplospory) essentially contains three components: *apomeiosis* (leading to formation of unreduced embryo sac); *parthenogenesis* (development of embryo without fertilization); and *functional endosperm development* (autonomous or pseudogamous) (Nogler, 1984a). These three components are linked functionally to generate apomictic seeds. Apomeiosis leads to the formation of meiotically unreduced embryo-sacs that contain egg cell, and polar nucleus/nuclei with sporophytic chromosome number (2*n*). The 2*n* egg cell then develops parthenogenetically (2*n* + 0 = 2*n*) to generate a 2*n* embryo. Embryogenesis is followed with development of endosperm either through fertilization of unreduced polar nucleus/nuclei (pseudogamy) or without fertilization (autonomous). This pathway of apomictic seed formation is represented as *apomeiosis:parthenogenesis:functional-endosperm development* (Asker and Jerling, 1992). The biological functions of the individual components and the progression from one stage to the next is presently under intense investigations (Grimanelli, 2012; Schmidt et al., 2015; Mirzagadheri and Horandl, 2016; Bocchini et al., 2018; Juranic et al., 2018).

### Partitioning Apomixis Components: Principle and Consequences

The apomixis components were believed to be strictly under control of “one major locus,” eventually generating an apomictic phenotype, in the majority of the agamic species (Savidan, 2000; Ozias-Akins and van Dijk, 2007). Accordingly, breeding strategies and molecular studies were designed for cultivar development, understanding the mechanism, mutagenesis and traits-introgression from related wild species. Occasional deviations from expected phenotypic frequencies and ploidies were considered as spontaneous off-types (Muntzing and Muntzing, 1943; Asker, 1980; Nakagawa and Hanna, 1990; Berthaud, 2001; Caceres et al., 2001). However, recent studies supported the fact that at-least in some of the apomictic taxa (see subsequent sections), the three apomixis components viz. apomeiosis, parthenogenesis and functional endosperm development may be uncoupled (Barcaccia and Albertini, 2013). Contrary to the “one locus” theory, the partitioning principle suggests apomixis under the control of three distinct genetic determinants, each controlling an individual component, and recombination between them possible. These recombinants have been isolated phenotypically in many apomictic species and the molecular principles (molecular markers, structural and functional genomics) underlying the mechanism are being investigated (Noyes, 2006; Zavesky et al., 2007; Kaushal et al., 2008; Koltunow et al., 2011; Conner et al., 2013).

**FIGURE 1:**
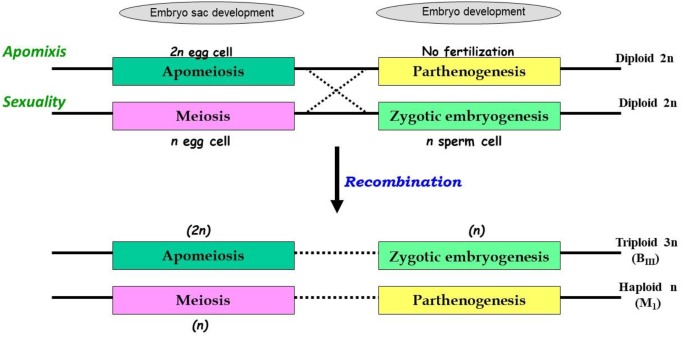
Consequences of partitioning apomixis components leading to formation of triploids (3*n*) and haploids (1*n*) in the progeny of diploids (2*n*), through B_III_ and M_1_ pathways. Apomixis is achieved when an unreduced egg cell (in apomeiotic embryo-sac) develops through parthenogenesis, however, in sexually reproducing plants meiotically derived haploid egg cell fertilizes with a haploid sperm cell. In both cases, diploid status (2*n*) of embryo is conserved. In presence of genetic determinants for both these functions, as in heterozygotes, recombination between these elements may occur. Consequently, apomeiotic egg cell might require fertilization for embryo development leading to triploid (2*n* + *n* = 3*n*; B_III_) progeny. Alternatively, a meiotically derived haploid egg cell acquires the parthenogenetic capacity and develops without fertilization, leading to formation of haploid (1*n* + 0 = 1*n*; M_1_) progeny.

The uncoupling may lead to newer combinations of partitioned apomixis components during the seed development process. As stated earlier, generation of clonal embryos relies on operating the *apomeiosis:parthenogenesis* pathway (2*n* + 0) and this functional linkage is necessary to maintain ploidy and clonality. However, as a consequence of uncoupling of apomixis components, the functional linkage between apomeiosis and parthenogenesis is lost, and thus apomeiotically derived unreduced egg cell (2*n*) loses the capacity of parthenogenesis and requires fertilization with male gamete (1*n*) for embryo development, eventually leading to the formation of a triploid embryo (2*n* + 1*n* = 3*n*). This recombined pathway may be represented as *apomeiosis:zygotic-embryogenesis*. Alternatively, in a typical sexually derived egg cell (1*n*), requirement of fertilization for embryo development is lost/replaced by parthenogenesis and the resultant embryo develops without fertilization yielding a haploid embryo (1*n* + 0 = 1*n*), following a *meiosis:parthenogenesis* pathway (Figure 1). Triploids derived through 2*n* + *n* hybridization are termed as B_III_ hybrids and the haploids (1*n* + 0) as M_1_ plants (Rutishauser, 1948). Similarly, sexually derived diploids and obligate apomicts are designated as M_II_ and B_II_, respectively (Rutishauser, 1948; Aliyu et al., 2010). Broad categories of seed formation arising through partitioning events, in Polygonum-, Hieracium- and Panicum-type ES, is summarized in Table 1.

**Table 1:** Matrix showing possible categories of seeds and genomic contributions in three types of Embryo sacs (ES).

Embryo-sac type and features	Embryo development		Endosperm development
	Parthenogenetic	Zygotic	
*Polygonum* type;(Reduced ES, 8 nucleated)Egg Cell 1*n*; Polar nuclei (1*n* + 1*n* = 2*n*)	1:3^a^Em (1*m*:0*p*); En (2*m*:1*p*)^b^	2:3Em (1*m*:1*p*); En (2*m*:1*p*)	Pseudogamous
	1:2Em (1*m*:0*p*); En (2*m*:0*p*)	2:2Em (1*m*:1*p*); En (2*m*:0*p*)	Autonomous

Broad Category	**M_1_**	**M_II_**	

*Hieracium* type;(Unreduced ES, 8 nucleated);Egg Cell 2*n*;Polar nuclei (2*n* + 2*n* = 4*n*)	2:5Em (2*m*:0*p*); En (4*m*:1*p*)	3:5Em (2*m*:1*p*); En (4*m*:1*p*)	Pseudogamous
	2:4Em (2*m*:0*p*); En (4*m*:0*p*)	3:4Em (2*m*:1*p*); En (4*m*:0*p*)	Autonomous

Broad Category	**B_II_**	**B_III_**	

*Panicum* type;(Unreduced ES, 4 nucleated);Egg Cell 2*n*;Polar nuclei (2*n*)	2:3Em (2*m*:0*p*); En (2*m*:1*p*)	3:3Em (2*m*:1*p*); En (2*m*:1*p*)	Pseudogamous
	2:2Em (2*m*:0*p*); En (2*m*:0*p*)	3:2Em (2*m*:1*p*); En (2*m*:0*p*)	Autonomous

Broad Category	**B_II_**	**B_III_**	


*Genome constitution in embryo and endosperm are depicted from seeds originated from three types of ES (Polygonum-, Hieracium- and Panicum-type). Embryo development may be zygotic or parthenogenetic, while endosperm development may be pseudogamous or autonomous. ^*a*^Ratio of genomes in embryo:endosperm; ^*b*^Contribution of maternal (*m*) and paternal genomes (*p*) in embryo (Em) and endosperm (En)*.

Such partitioning events are largely believed to be consequence of recombination between apomixis components, however these are also influenced by modifiers and epialleles, and may show varied expressivity and penetrance (Noyes, 2006; Zavesky et al., 2007; Kaushal et al., 2008, 2018; Conner et al., 2013). This is expected in view of the suggested origin of apomixis via hybridization (maintaining a state of heterozygosity) (Nogler, 1984a; Carman, 1997; Talent, 2009). These “heterozygotes” may harbor genetic determinants for both apomixis and sexual reproduction and become amenable to uncoupling, owing to above mentioned factors. Similar heterozygous situation also prevails in progeny derived from experimental crosses between sexual × apomictic parents. As an illustration, haploid (M_1_) progeny between sexual and apomictic parents in *Potentilla collina* was recovered through “*parthenogenetic development of reduced ovules*,” whereby tendencies of formation of reduced gametes and the development of an egg cell without fertilization were derived from sexual and apomictic parent, respectively (Muntzing and Muntzing, 1943). Partitioning is also reported in experimental crosses between sexual and apomictic forms (intra- as well as inter-specific hybridization) in *Ranunculus*, *Panicum*, *Pennisetum, Cenchrus*, and *Poa* (Savidan, 2000; Matzk et al., 2001, 2005; Ozias-Akins and van Dijk, 2007; Kaushal et al., 2008; Barcaccia and Albertini, 2013).

Similarly, for the third component of apomixis, viz., functional endosperm development, the mode of formation (pseudogamous or autonomous) may also get modified as a consequence of partitioning. For example, induction of autonomous mode of endosperm development in otherwise pseudogamous species by acquiring additional genetic determinants or by removal of suppressors that restrict the proliferation of polar nuclei in absence of fertilization, or vice versa. Such modifications are reported in several apomictic species (e.g., *Taraxacum officinale*, *Panicum maximum, Hieracium* spp., etc.) and in mutants mimicking apomixis components in otherwise sexual crops (van Dijk et al., 2003; Bicknell and Koltunow, 2004; Kaushal et al., 2008; Schmidt et al., 2015; Brukhin, 2017). During apomixis, although clonal embryos are generated from all categories of apomeiotic ES, the genomic ratios in endosperm largely depends on the developmental category of ES. For example, genome ratios (embryo:endosperm) in seeds derived from Polygonum- and Panicum-type ES are 2Em:3En, while it is 2Em:4En or 2Em:5En in Hieracium-type ES derived through autonomous or pseudogamous development, respectively (Table 1). Relative genome contribution of maternal and paternal genomes in embryo and endosperm constitution and the imprinting effects are thereby being studied to gain an insight into key genetic and epigenetic factors for successful endosperm development. Diversity in endosperm development, molecular mechanisms and the constraints of endosperm imprinting effects, are some of the issues of recent investigations (Pupilli and Barcaccia, 2012; Gehring and Satyaki, 2017; Henderson et al., 2017; Brauning et al., 2018; Depetris et al., 2018).

It would be interesting to debate whether apomeiosis and parthenogenesis have independent origins (Horandl, 2009; Talent, 2009; Tilquin and Kokko, 2016). In many taxa, the independent occurrence for capacity to generate unreduced gametes or haploid parthenogenesis suggest their independent origin during evolutionary lineage, however, their recurrent occurrence over generations will either polyploidize them out of existence or lead to haploid sterility. Interestingly, a combination of these two components stabilizes the system by maintaining the ploidy state, in spite of their individual capacities to modify it. From an evolutionary perspective, it seems logical that linkage between the apomixis components is essential for survival and perpetuation of the species to maintain the hybridity and ploidy, overcoming the constraints of genomic imbalances and ploidy levels of parental species. Species demonstrating partitioned apomixis components are regarded as evolutionary young apomicts, as compared to the species where recombination is suppressed (Pupilli and Barcaccia, 2012; Hand and Koltunow, 2014).

In a strict sense, the manifestation of sexuality might occur at two stages during seed development: the formation of meiotic (or sexual ES) which allows meiotic progression to generate variability in the gametes (in obligate sexual or facultative individuals), as well as during fertilization between male and female gametes (syngamy), irrespective of the meiotic or apomeiotic origin of the ES (yielding B_II_/M_II_ or B_III_ hybrids, respectively). Interestingly, isolated apomixis components generate variability, and act as a driving force in evolving agamic species (Berthaud, 2001). The situation may be more complex in facultative individuals, as both sexual and apomeiotic factors are present in the same genotype, though with different extensions (Delgado et al., 2016; Kaushal et al., 2018).

### Detection Methods to Identify Partitioned Apomixis Components

Modifications arising due to partitioning of apomixis components (ploidy of the progeny and mode of endosperm development) can be identified utilizing the characterization of embryo-sacs and ploidy estimation of the embryo of the progeny (Crane, 2001).

**FIGURE 2:**
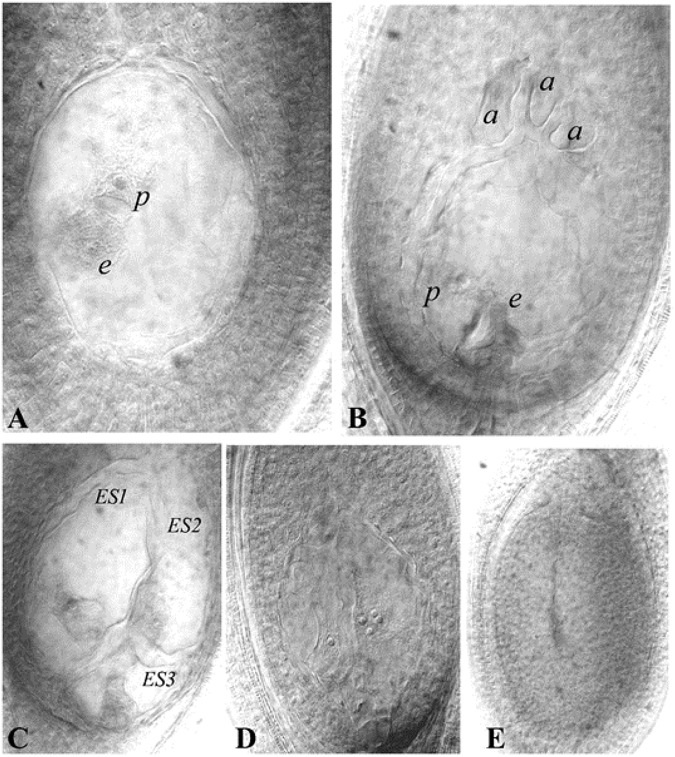
Representative ES of guinea grass (cleared ovules). **(A)** Aposporous ES, **(B)** Sexual (or meiotic) ES, **(C)** Multiple ES (three ES seen), **(D)** Ovule showing proliferating polar nuclei in absence of pollination, as an indicator of AED, a cluster of four nuclei is visible in one plane, **(E)**: Ovule showing an aborted ES. *e-* egg cell, *p*-polar nucleus, *a*- antipodals, *ES-*Embryo sac. Reprinted by permission from the Springer Nature: Kaushal et al. (2018).

Histological differences between reduced (e.g., Polygonum-type: 8-nucleated sexual types) and unreduced ES (e.g., 4-nucleated Panicum-type) have been utilized to characterize the presence of apomeiosis/meiosis. Methods to analyse ES structure development within the ovule have been modified from classical sectioning procedures to more rapid callose deposition tests (Peel et al., 1997; Tucker et al., 2001) and ovule clearing techniques (Young et al., 1979; Herr, 2000; Crane, 2001). Cleared ovules are now extensively utilized to characterize mode of ES formation, quantification of apospory and abortive ES, as well as to observe autonomous endosperm development (AED) (Figure 2).

The potential for parthenogenesis can be tested using “auxin test” (or auxin-induced parthenocarpy) (Matzk, 1991). Auxins replace the endosperm effect, thereby allowing initial development of embryo in the absence of endosperm, provided parthenogenesis genes are available. Auxin test has been successfully utilized to identify parthenogenesis potential in *Poa* spp., *Hypericum* spp., wheat-salmon system (Matzk et al., 2007) and *Dichanthium annulatum* (Gupta et al., 1999).

**FIGURE 3:**
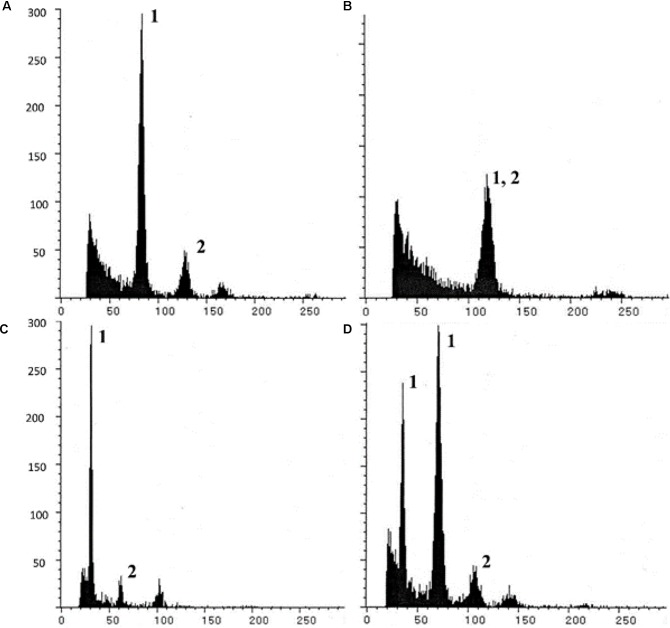
Illustrative Single seed-FCSS histograms showing various progeny classes obtained in *P. maximum*, exhibiting **(A)** 2*n* embryo (B_III_ or M_1_ origin; with 2Emb:3End genome ratios); **(B)** 3*n* embryo (B_III_ seed; 1Emb:1End ratio; **(C)** 1*n* embryo (M_1_ seed; 1Emb:3End ratio); and **(D)** a seed with twin embryos with *n* (M_1_ origin) and *2n* genomes and sharing common endosperm. In histogram, *x* axis- relative fluorescence, *y* axis- number of nuclei; Peaks 1 and 2 represent embryo and endosperm peaks, respectively, other unmarked peaks arise from endo-polyploidization events of embryo/endosperm cells. Reprinted by permission from the Springer Nature: Kaushal et al. (2018).

Triploids (B_III_) and haploids (M_1_) were identified through classical chromosome-counting methods (Asker, 1980), and more recently using flow cytometric measurements of sporophytic DNA (Aliyu et al., 2010; Conner et al., 2013; Kaushal et al., 2018). The principle of flow cytometry was also utilized to develop a highly efficient and rapid screen, described as the Flow Cytometric Seed Screen (FCSS) (Matzk et al., 2000), which analyzes relative DNA contents of embryo and endosperm cells (from single/bulked matured seeds) to estimate their ploidies (Figure 3). When appropriately supplemented with information on the mode of ES development, ploidy of contributing male gametes (reduced/unreduced) and mode of endosperm development (autonomous/pseudogamous) can also be estimated, thereby enabling reconstruction of the possible reproductive pathways of seed formation. FCSS has been successfully implemented in confirming partitioning effects in diverse aposporouos and diplosporous apomicts, such as *Brachiaria* spp., *C. ciliaris*, *Panicum maximum*, *Boechera* spp., *Hypericum* spp., *Poa* spp., *Tripsacum dactyloides*, *Hieracium* spp., *Paspalum simplex*, *Onosma* spp., *Rosa canina*, *Capsella bursa-pastoris*, *Crataegus* spp., *Ranunculus auricormus* complex, etc., (reviewed in Krahulcova and Rotreklova, 2010; Kolarcik et al., 2018). FCSS also provides an opportunity (over progeny analysis) to analyze those proportions of seeds that might fail to germinate, owing to disturbed embryo:endosperm ratios, hence providing a better estimate of the partitioning events (Kaushal et al., 2008; Conner et al., 2013).

In addition to above analytical techniques, molecular markers tightly linked to individual components are also being utilized to identify partitioning events (see later sections for details on molecular markers) (Pupilli and Barcaccia, 2012; Barcaccia and Albertini, 2013; Conner et al., 2013; Hand and Koltunow, 2014; Brukhin, 2017).

### Partitioning Apomixis Components in Natural Apomictic Systems

Genetic analysis and utilization of efficient screening techniques led to identification of apomictic species with possible recombination between apomixis components, both in aposporous apomicts such as *R. auricormus* (Nogler, 1984b), *Poa pratensis* (Albertini et al., 2001; Matzk et al., 2005), *Hieracium* spp. (Catanach et al., 2006), *Panicum maximum* (Kaushal et al., 2008) *Hypericum perforatum* (Matzk et al., 2001; Schallau et al., 2010), as well as diplosporous apomicts, such as *Erigeron annus* (Noyes and Rieseberg, 2000) and *T. officinale* (van Dijk and Bakx-Schotman, 2004) (reviewed in Krahulcova and Rotreklova, 2010; Pupilli and Barcaccia, 2012; Barcaccia and Albertini, 2013).

Cytogenetical and genetic mapping studies demonstrated the possibility and consequences of recombination between the apomixis components. It has been suggested that the recombination between apomeiosis and parthenogenesis (and/or functional endosperm development) may not be mutually exclusive, along with involvement of minor loci or modifiers in governing the phenotype (Barcaccia et al., 2006, 2007). As an illustration, in *H. perforatum*, most parthenogenetic plants were aposporic, however, several aposporic plants were non-parthenogenetic and recombinants for parthenogenesis were 10-folds higher than recombinants for apospory (Schallau et al., 2010). Similarly, in apomictic *P. maximum* germplasm accessions, parthenogenesis was uncoupled from apospory in about 26% of cases (Kaushal et al., 2008). Similar results were also reported in *P. pratensis* and *H. perforatum* (Matzk et al., 2001, 2005). However, there are a couple of reports on complete and independent expression of apomeiosis, including an apomeiotic non-parthenogenetic inter-specific hybrid between two sexual diploid species viz., *Pennisetum glaucum* and *P. orientale* (Kaushal et al., 2010), LOA and LOP mutants in *Hieracium* (Koltunow et al., 2011) and an ASGR recombinant in *C. ciliaris* (Conner et al., 2013).

As the expression of apomixis and its components is largely affected by genotype and hybridity, identification of partitioning events relies on exploring sufficiently large and diverse germplasm collections, including (experimental) hybrids between sexual and apomictic parents. A survey of a sufficiently large germplasm base identified the occurrence of partitioning in *P. pratensis* (Matzk et al., 2005), *H. perforatum* (Matzk et al., 2001) and *Pancium maximum* (Kaushal et al., 2008), as also in experimental hybrids, e.g., in *R. auricomus* (Nogler, 1984b), *P. collina* (Muntzing and Muntzing, 1943), *P. maximum* (Kaushal et al., 2008) and *C. ciliaris* (Conner et al., 2013). Although in *Paspalum*, parthenogenesis and apospory were reported to be inherited together (Pupilli and Barcaccia, 2012), in inter-varietal crosses between sexual and apomictic parents, B_III_ hybrids were reported to occur (though in low frequency) in most of the apomictic progenies, and the uncoupling between apospory and parthenogenesis occurred among up to 50% cases (Caceres et al., 2001). Similarly, in *P. maximum*, wherein apomixis was believed to be monogenic (Savidan, 2000), uncoupling events were demonstrated in a wide scale screening of 669 genotypes (including a global germplasm collection), as well as in experimental hybrids (Kaushal et al., 2008, 2009). Recently, uncoupling of apomixis components has also been reported in *C. ciliaris* progenies obtained from sexual × apomictic lines utilizing FCSS and molecular markers analysis (Conner et al., 2013; I, ndian Grassland and Fodder Research Institute [IGFRI]2013). These reports suggest that among many crops un-reported for partitioned apomixis components, a greater diversity in reproductive development regarding uncoupling of apomixis components is expected to be discovered by screening a larger and more diverse germplasm base, including the crosses between parents with contrasting reproductive capacities, and utilizing more efficient screening techniques.

## Genetic Regulation of Partitioned Apomixis Components

### Induction and Inheritance of Apomixis Components

Experimentally, hybridization and polyploidy were attempted to test their potential to induce individual apomixis components, owing to the fact that these two are major contributory forces for origin of apomixis. Reports on *de novo* appearance of apomeiosis component through hybridization and/or polyploidization are more frequent, as compared to parthenogenesis and modification in endosperm development (reviewed in Mason and Pires, 2015; Kreiner et al., 2017).

From an apomixis perspective, induction of apomeiosis (apospory) has been reported as early as 1967 in certain hybrids of *Sanguisorba* (Nordborg, 1967) and in intergeneric hybrid *Raphanobrassica* (Ellerstrom and Zagorcheva, 1977; Asker, 1980). However, empirical results on the appearance of spontaneous apospory by inter-varietal or inter-specific hybridization between two sexually reproducing species, which eventually modified the mode of embryo-sac formation, have been recently reported in *Pennisetum* (Kaushal et al., 2010) and *R. auricomus* (Hojsgaard et al., 2014a). An interspecific hybrid (2*n* = 16, genome *GO*) between two diploid and sexually reproducing species (Polygonum-type ES), viz. *P. glaucum* (2*n* = 14; *GG*) and *P. orientale* (2*n* = 18; *OO*), showed a transition from obligate sexuality (Polygonum-type ES) to apospory (>83% Panicum-type aposporous ES). Parthenogenesis was completely omitted in this plant, and it produced all B_III_ hybrids (2*n* = 23; *GGO*) when backcrossed with *P. glaucum*. The capacity for apomeiosis and zygotic embryogenesis was stable and inheritable in this hybrid, although a dosage effect was observed whereby upon adding sexual genome(s) from *P. glaucum* or apomictic genome from *P. squamulatum*, the expressivity of apospory was reduced or enhanced, respectively. The hybrid (*GO*) also demonstrated *de novo* induction of AED (proliferation of polar nuclei), suggesting the induction of this component is also affected by hybridity. Similarly, sterility effects were overcome in interspecific and inter-ploidy crosses in *Ranunculus* by resorting to spontaneous apospory in mode of ES formation, eventually forming viable triploid seeds (Hojsgaard et al., 2014a). A novel phenomenon was also described for induction of apomeiosis through second division restitution in interspecific cross between *Saccharum officinarum* and *S. spontaneum*, whereby formation of a 2*n* female gamete was triggered by the male gamete (Hermann et al., 2012). The induction is dependent on ploidy of *S. sponateum* as the male gamete in a dose-dependent manner and possesses the potential to be utilized for *in vivo* production of doubled haploids in intergeneric crosses. The induction of apomeiosis is known to be affected by hybridity and/or polyploidy, explainable on the basis of HFA theory (Carman, 1997) as well as epigenetic reprogramming of the genes involved in embryo-sac and endosperm development (Grimanelli, 2012; Kreiner et al., 2017; Hojsgaard, 2018).

Reports on induction of parthenogenesis through interspecific hybridization are rare, although inter- or intra-specific hybridization has been used to trigger haploidy via alternative pathways, such as uniparental genome elimination, utilizing genetic and cytogenetic stocks and alloplasmic cytoplasms (reviewed in Forster et al., 2007; Ishii et al., 2016). In apomictic systems, parthenogenesis component is generally contingent upon apospory or diplospory (Ozias-Akins and van Dijk, 2007). It is easier to partition it from apomeiosis, however, independent recurrent parthenogenesis is rarely naturally reported in plants, though it has been achieved experimentally (e.g., *lop* mutants in *Hieracium*; PsBBML in *Pennisetum*) (Koltunow et al., 2011; Conner et al., 2015; Mirzagadheri and Horandl, 2016).

Inheritance studies showed dominant inheritance of the partitioned apomixis components, however, with variable penetrance and expressivity, and were influenced by genotype and ploidy (reviewed in Ozias-Akins and van Dijk, 2007; Pupilli and Barcaccia, 2012; Barcaccia and Albertini, 2013; Hand and Koltunow, 2014). In *P. pratensis*, a multigene inheritance model has been proposed (Matzk et al., 2005), however, inheritance of parthenogenesis (*PARTH1*) as a dominant single gene was also proposed (Porceddu et al., 2002). Apomixis in *T. officinale* is under the control of two independent loci, one for diplospory (*DIP*) and the another for parthenogenesis (*PAR*) (Vijverberg et al., 2004). Similarly, two independent dominant loci models have been proposed in diplosporous *Erigeron annuus*, one for diplospory (*D*) and the other (*F*) for both parthenogenesis and AED (Noyes et al., 2007). Three dominant loci, viz., LOA, LOP and AutE control individual apomixis components in *Hieracium* sugenus *Pilosella* (Koltunow et al., 2011; Henderson et al., 2017).

### Candidate Genes for Individual Components

The fact that apomictic and sexual systems share a common network of gene actions during seed development (Hand and Koltunow, 2014), supplemented the efforts on identification of genes mimicking apomixis components in sexual systems. Mutants of these genes/genomic regions from sexual systems have been identified to exhibit apomixis components, and those involved in essential functions during megasporogenesis, meiosis initiation and progression, megagametogenesis, embryogenesis and endosperm development (e.g., *DYAD*, *SWI1*, *Elongate1*, *SERK*, *ARG*, *MiMe* sets, *AGO*, *DMT*, *hap*, *BBM*, *FIE*, *MEA*, *DME*, etc.) (reviewed in Pupilli and Barcaccia, 2012; Barcaccia and Albertini, 2013; Schmidt et al., 2015; Brukhin, 2017). Transcriptome analysis involving ovular tissues during apomixis or sexual process have been compared in aposporous apomicts (e.g., *Brachiaria brizantha*, *Pennisetum* interspecific hybrids, *C. ciliaris*, *P. maximum*, *Paspalum notatum*, *H. perforatum*) and diplosporous apomicts (e.g., *Boechera*), and differentially expressed genes were identified (Reviewed in Conner and Ozias-Akins, 2017).

Additionally, detailed molecular analysis of genomic regions governing apomixis in natural apomictic systems led to the identification, characterization and isolation (in several cases) of key genes involved in apomictic reproduction *per se* or its components. These include genes controlling apomeiosis, such as *APOLLO* (Apomixis linked locus; *Boechera* spp.) (Corral et al., 2013), *HAPPY, HpARI* (*ARIADNE7*; *H. perforatum*) (Schallau et al., 2010), those controlling parthenogenesis, such as *ASGR-BBML* (Apospory Specific Genomic Region-Baby Boom; *Pennisetum squamulatum*) (Conner et al., 2015); as well as that modulating endosperm development, such as PsORC3a (Origin Recognition Complex; *P. simplex*) (Siena et al., 2016) and *AutE* (AED; *Hieracium* subgenus *Pilosella* species) (Henderson et al., 2017). Promising results towards introduction of the parthenogenesis component of apomixis has been provided by utilizing *PsASGR-BBML* gene, which successfully developed parthenogenetic haploids in sexual crops such as pearl millet, rice and maize (Conner et al., 2015, 2017), and is reported to be conserved across Paniceae species (Worthington et al., 2016).

### Factors Affecting Uncoupling and Expression of Partitioned Apomixis Components

Partitioning of apomixis components and their expression have been found to be largely influenced by genotypic effects, however, they are also affected by ploidy levels and dosage effects, as well as stress and environmental factors. Modifying elements present in the genetic background have also been presumed to modulate the expressivity of apomixis components (Koltunow et al., 1998; Bicknell et al., 2000; Hand et al., 2015), mostly by epigenetic regulatory networks (Curtis and Grossniklaus, 2008; Galla et al., 2017; Bocchini et al., 2018).

In apomictic plants, genotypic effects were identified to be more profound than ploidy effects in determining the mode of reproduction, as well as penetrance and expressivity of the component traits. Although modification of ploidy levels effect partitioning, it is largely found to be genotype-dependent (Burson et al., 2002; Matzk et al., 2005; Kaushal et al., 2008, 2018; Krahulcova and Rotreklova, 2010; Sharbel et al., 2010; Krahulec et al., 2011; Voigt-Zielinski et al., 2012; Delgado et al., 2014; Noyes and Wagner, 2014). Higher ploidy may accumulate the relative doses of the *apomeiotic-* or *sexual-factors*, which in turn affects the eventual expression of the trait, especially in facultative genotypes. Such dosage effects on expression of apomeiosis have been reported in apomicts, such as *R. auricomus*, *Erigeron* interspecific hybrids, *Paspalum rufum*, *P. maximum*, *Pennisetum* interspecific crosses and *Pilosella* spp. (Nogler, 1984b; Noyes, 2005; Kaushal et al., 2008, 2010; Krahulcova et al., 2011; Delgado et al., 2016). Interestingly, an enhancement in sexuality (or reduction in apospory) has been reported with rise in ploidy in a *P. maximum* ploidy series (2*n* = 6*x* to 11*x*) (Kaushal et al., 2018).

In general, occurrence of the *apomeiosis:zygotic-embryogenesis* pathway (leading to B_III_ hybrids) is reported more frequently than the *meiotic:parthenogenesis* pathway (M_1_, di/poly-haploids) (Bicknell et al., 2003; Aliyu et al., 2010; Hojsgaard et al., 2014a; Schinkel et al., 2017; Klatt et al., 2018a). However, B_III_ formation is found largely to be genotype-dependent and ploidy level has little effect on the expression of partitioned apomeiosis. In fact, partitioning and formation of B_III_ hybrids have been recently reported in diploid individuals in agamic complexes of *Boechera* and *Ranunculus* (Aliyu et al., 2010; Hojsgaard et al., 2014a; Schinkel et al., 2017; Klatt et al., 2018b; Barke et al., 2018). On the other hand, expression of the parthenogenesis component is highly influenced by the ploidy variations exhibiting high positive correlation with increasing ploidy (Kaushal et al., 2009; Aliyu et al., 2010; Noyes and Wagner, 2014). Recently, a strong relationship was identified between rise in ploidy and frequency of haploid production in plants with 6*x* ploidy and more (2*n* = 6*x* till 2*n* = 11*x*) in an exhaustive ploidy series of *P. maximum* (Kaushal et al., 2018), suggesting that these “*parthenogenetic factors*” may also act in a dosage dependent manner. Different effects of changes in ploidy level on expression of apomeiosis and parthenogenesis suggest existence of different mechanisms controlling these two traits (reviewed in Sokolov et al., 2008). Haploids (or poly-haploids), resultant of haploid parthenogenesis are rare in diploid plants, explainable on the basis of minimum gene-dosage model, segregation-distortion model, or gametophyte-expressed lethal model (reviewed in Bicknell and Koltunow, 2004; Talent, 2009; Cosendai and Horandl, 2010). From an evolutionary perspective, these partitioned components act as a natural phenomenon to enrich the species diversity and speciation through polyploid-polyhaploid-polyploid cycles, as demonstrated in *D. annulatum* (de Wet, 1968), *P. maximum* (Savidan and Pernes, 1982), *Eragrostis curvula* (Mecchia et al., 2007), *Boechera* spp. (Aliyu et al., 2010) and *Erigeron* spp. (Noyes and Wagner, 2014).

In addition to the above factors (genotype and ploidy), environmental stresses, such as higher elevations, extreme temperatures and edaphic factors, seasonal variations, nutrition, herbivory and diseases, as well as pollination timings, are also known to affect the expressivity and penetrance of apomeioisis and parthenogenesis traits (Cosendai and Horandl, 2010; Mason and Pires, 2015; Schinkel et al., 2016; Shah et al., 2016; Kreiner et al., 2017; Rodrigo et al., 2017; Kirchheimer et al., 2018; Klatt et al., 2018b). A role of stress hormone signaling has been proposed for initiating such responses and has been studied in biochemical as well as evolutionary perspectives (Koltunow et al., 2001; Polegri et al., 2010; Horandl and Hadacek, 2013). In-fact it would be interesting to identify a stress-activated molecular switch that can trigger the expression of apomixis components or vice versa. Timing of pollination is also reported to be a factor affecting frequency of B_III_ hybridization events (Martinez et al., 1994; Burson et al., 2002; Espinoza et al., 2002).

Recombination between components may also modify mode of endosperm development in a genotype dependent manner. Such modifications are largely identified to be genotype-dependent, with little effect of the ploidy levels, and modulated by still unknown regulatory factors (Li et al., 2014; Hand et al., 2015; Gehring and Satyaki, 2017; Henderson et al., 2017; Kaushal et al., 2018).

## *Panicum Maximum* as a Model System to Study Partitioning of Apomixis Components

*Panicum maximum* Jacq. (syn. *Megathyrsus maximus*, family: Poaceae, subfamily: Panicoideae, tribe: Paniceae), commonly known as guinea grass, is a suitable system for polyploidy and apomixis research. It is a tall, high yielding, nutritious, perennial, high seed setter, and multi-cut forage grass, adapted to humid, semi-arid and arid environments. This crop possesses substantial variability in morphology, breeding and agronomic traits (Malaviya, 1998; Kaushal et al., 1999; Sukhchain, 2010), and the global germplasm diversity has been characterized for cytological, biochemical and molecular features (Jain et al., 2003, 2006; Ebina et al., 2007; Chandra and Tiwari, 2010; Sousa et al., 2011).

Naturally occurring forms are predominated by apomictic tetraploid cytotypes (2*n* = 4*x* = 32), with occasional reports of sexual diploids (2*n* = 16) and facultative hexaploids (2*n* = 48) (Savidan, 2000; Jain et al., 2003; Kaushal et al., 2008). Sexually reproducing tetraploid lines are also reported to occur naturally as well as in experimentally induced polyploids (Nakajima et al., 1979; Hanna and Nakagawa, 1994). It has a smaller genome size (ca. 500 Mbp) and ca. 0.9 pg sporophytic DNA content (in diploid strains) (Akiyama et al., 2008; Kaushal et al., 2009). Availability of sexual as well as apomictic forms within the same ploidy level makes it a suitable system to generate desired populations to undertake inheritance and molecular biology studies.

The mode of seed formation is *apospory:parthenogenesis:pseudogamous-endosperm* in apomictic forms, while sexual genotypes produce seeds by syngamy of reduced male and female gametes, followed by pseudogamous endosperm development. Apomeiosis is characterized by Panicum-type aposporous ES (2 synergids, 1 egg cell, 1 polar nucleus; all 4-nuclei are unreduced), while sexual lines exhibit typical Polygonum-type reduced ES (2 synergids, 1 egg cell, 2 polar nuclei, 3 antipodals; all eight nuclei are reduced). Anatomical differences between aposporous and sexual ES permit rapid analysis for identification of mode of reproduction in germplasm and segregating populations (Nakagawa, 1990).

A dominant single gene model for controlling apomixis in guinea grass has been widely accepted (Savidan, 1980, 2000; Ebina et al., 2005), governing apomixis phenotype in simplex condition (*Aaaa*). Development of aposporous ES has been extensively studied cytologically and ultra-structurally (reviewed in Chen and Guan, 2012). Although still to be genome-sequenced, this crop is rich in available genomic resources. Molecular markers (RAPD, RFLP, AFLP, SSR and ESTs) linked to apomixis have been developed and the aposporous linkage group has been constructed (Ebina et al., 2005; Bluma-Marques et al., 2014). Expressed sequence tags (ESTs) led to identification of aposporous ovary-specific genes (Yamada-Akiyama et al., 2009). Richness of molecular resources in this crop is further strengthened by availability of extensive genomic databases in its close relative *Panicum virgatum* (switch grass) (Sharma et al., 2012; Zhang et al., 2013).

An *Apomixis Specific Gene* (*ASG-1*), that showed stage specific expression in developing buds of apomictic types only, has been identified and characterized through comparative gene expression analysis (Chen et al., 1999, 2005). Transcriptome data has been generated comparing the gametogenesis stages between apomictic and sexual forms (Radhakrishna et al., 2018). Recently, irradiation-induced deletion mutants for the apomixis, controlling genomic region in tetraploid guinea grass, showed loss-of-apomixis phenotype and replaced aposporous (Panicum-type) ES with sexual type (Polygonum-type) (Takahara et al., 2016).

In contrast to a general understanding of apomixis under monogenic control, a wide scale screening of guinea grass germplasm suggested multigene control of the trait. Uncoupling of the apomixis components was demonstrated in more than 67% of the global germplasm accessions, suggesting frequent occurrence of recombination between apomeiosis and parthenogenesis components (Kaushal et al., 2009). Germplasm lines with high B_III_ and M_1_ formation were also identified. Reproductive diversity for seed formation estimated through reconstruction of reproductive pathways (utilizing ES and FCSS analysis), in tetraploid and hexaploid guinea grass lines, suggested that the three components (apomeiosis, parthenogenesis and functional endosperm development) recombined freely and all phenotypic classes expected from such recombination events were recovered (Figure 1 and Table 1) (Kaushal et al., 2008). Identification of certain modified pathways e.g., presence of two polar nuclei in aposporous ES fusing prior to fertilization, and fusion of only one polar nucleus in a sexual ES, provides the opportunity for better insights into seed development processes. The flexibility of guinea grass to demonstrate aposporous and sexual ES, parthenogenetic and zygotic embryo development, and pseudogamous and AED (in ovules and matured seeds) offer advantages to understand the interaction effects arising due to recombination between these apomixis components.

**FIGURE 4:**
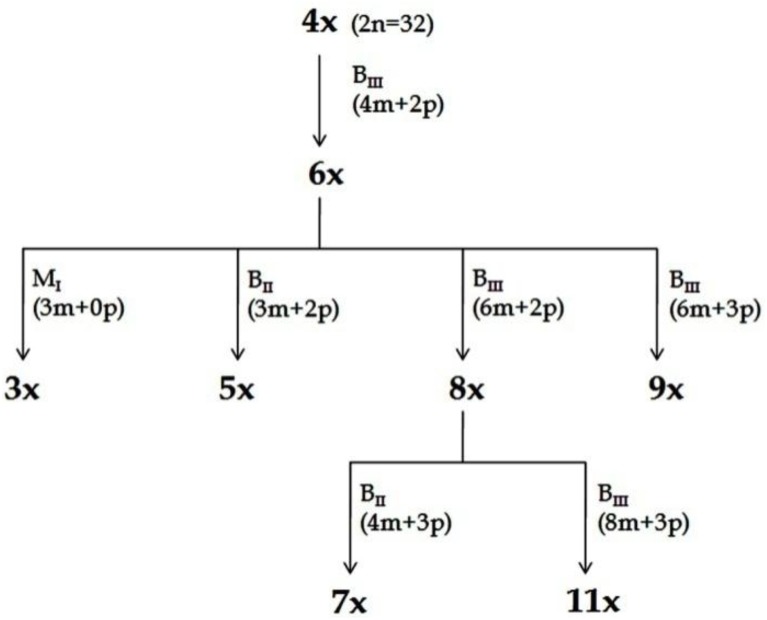
Scheme for production of ploidy series (Kaushal et al., 2009, 2015b). Plants representing different ploidies viz., 3*x*, 4*x*, 5*x*, 6*x*, 7*x*, 8*x*, 9*x*, and 11*x*, were generated from a single 4*x* progenitor through HAPA. The recovery of plants with specified ploidy and their pathways of formation (M_1_, B_II_ or B_III_) is depicted. Information in parenthesis shows maternal (*m*) and paternal (*p*) genomic contribution. In all cases depicted here, male gamete was always reduced, while female gamete might be reduced or unreduced, and the embryo development may be through parthenogenesis or fertilization dependent. Reprinted by permission from the Springer Nature: Kaushal et al. (2018).

**FIGURE 5:**
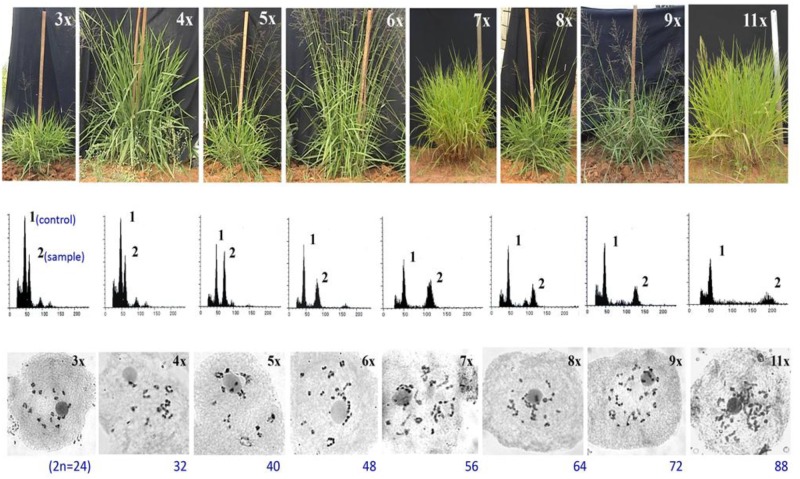
Morphological, flow cytometric and meiotic chromosomes characterization of a ploidy series in guinea grass, represented by 3*x*, 4*x*, 5*x*, 6*x*, 7*x*, 8*x*, 9*x*, and 11*x* cytotypes, generated through HAPA. *Upper lane*: morphological features of plants representing different ploidies. Vertical scale represents 100 cm height; *Middle lane*: Flow cytometric histograms from plant sample alongwith internal control, obtained from leaf tissues of plants representing various ploidies represented in upper lane, respectively. A triploid (3*x*) plant was used as the internal control, represented by peak 1 in all FCM histograms; peak 2 was of the sample. Smaller peaks arise from G2 phase cells. Leaves of 4*x* plant were used as the internal control for 3*x*, and hence the histograms of 3*x* and 4*x* plants were same; *Lower lane*: meiotic chromosome configurations of plants representing ploidies 3*x* till 9*x* and 11*x*. Adapted and modified with permission from Kaushal et al. (2009). Reprinted by permission from John Wiley and Sons: Kaushal et al. (2009).

Consequences of partitioned apomixis components, leading to the formation of triploids (3*n*, B_III_ hybridization) and/ or haploids (1*n*, M_1_ progeny), was utilized to develop a Hybridization-supplemented Apomixis-components Partitioning Approach (HAPA) for ploidy manipulations without using any chemical agent or *in vitro* processing. Utilizing HAPA, an exhaustive ploidy series has been developed from a single 4*x* (2*n* = 32) progenitor, represented by 3*x*, 4*x*, 5*x*, 6*x*, 7*x*, 8*x*, 9*x*, and 11*x* cytotypes (Kaushal et al., 2009, 2015b) (Figure 4, 5). Such an exhaustive ploidy series offers an excellent system to understand ploidy regulated trait expression with respect to apomixis and its component traits. Male fertility is maintained at all these ploidy levels, providing a better scope for genetical and breeding experiments (Kaushal et al., 2018). Guinea grass is, thus, found to possess extraordinary flexibility to accommodate extreme genome dosage (2*n* = 2*x* till 11*x*), chromosome numbers (2*n* = 2*x* = 16 till 2*n* = 11*x* = 88) and sporophytic DNA content (1.8 pg to 5.0 pg), and is still capable of producing functional female gametes (both reduced and unreduced) and male (mostly reduced) gametes.

Ploidy effects on overall expression on apomixis revealed that the eventual phenotype depends on relative doses of apospory and parthenogenesis factors (Kaushal et al., 2018). Intriguingly, the proportion of facultatively reproducing progenies increased with the enhancement in ploidy levels. The phenotypic expression of partitioned apomictic components demonstrated B_III_ hybridization and AED to be less effected by the change in ploidy and were mostly dependent on genotypic effects. However, formation of M_1_ progeny was highly affected by a rise in ploidy, however, appeared only in plants with 6*x* ploidy or more (Combes, 1975; Kaushal et al., 2008, 2018). Availability of genotypes with similar ploidy level but contrasting capacities for partitioned components (extreme high or low frequency of formation of B_III_ or M_1_ progeny) are important resources to identify differentially expressed genes governing these partitioned components.

Endosperm development in guinea grass is intriguing, considering the fact that the typical 2:3 genome ratios is conserved in the embryo and endosperm of matured seeds by virtue of modification in the embryo-sac, which is a 4-nucleated Panicum type (as discussed earlier). However, it shows extraordinary flexibility in tolerating excessive deviance from typical 2em:3end genome ratios, as well as maternal and paternal genome contributions in developing embryos and endosperms. Em:End genome ratios tolerated are 2:3 (in B_II_/M_II_ progenies), 1:1 (in B_III_) and 1:3 (in M_1_) (Table 1). As an illustration, a 11*x* (2*n* = 88) plant will have 11em:16.5end and ≈16.5em:16.5end genome ratio in typical apomictic and B_III_ seed, respectively. Successful recovery of seeds, representing almost all categories (B_II_/M_II_, B_III_ and M_1_) from plants representing ploidy series (see Table 1), suggests that EBN and endosperm imprinting constraints are largely relaxed in this crop (Kaushal et al., 2018). Recovery of fertile seeds from such diverse categories is also important for studying nucleo-cytoplasm as well as embryo-endosperm interactions and the ovule molecular-machinery capable of bearing such high genomic content.

The diversity in pathways of seed formation, availability of plants representing different modes of reproduction and different ploidy levels, male fertility in plants with higher ploidies and successful recovery of seeds at extremely higher ploidies (some of them expressing B_III_ and M_1_ hybridization), all make this crop a potentially useful system for undertaking investigations in apomixis genetics and breeding, as well as cytogenetical and molecular studies on partitioned apomixis components.

## Utilization of Partitioned Apomixis Components

Understanding the partitioning phenomenon as well as utilization of the partitioned apomixis components have experimental and applied consequences. The foremost importance is towards a better understanding of apomeiotic ES development as well as elucidating parthenogenetic factors responsible for autonomous egg cell development, as the plant material polymorphic for differential capacities for these components is now available (Koltunow et al., 2011; Sahu et al., 2012; Hojsgaard et al., 2014a). It also may shed light on embryo-endosperm interactions especially for EBN and endosperm imprinting effects, chromatin dynamics, evolution of components, and more specifically the progression of the components in apomixis process.

Partitioning apomeiosis from parthenogenesis also allows for generation of variability, because the two stages of variability generation, viz., meiosis and fertilization, respectively, are rendered operational. Such possibilities eventually defy the perception considering apomixis as evolutionary dead-end and road-block to plant breeding (Darlington, 1939; Grant, 1981). Variability has been successfully generated through addition of genomes utilizing B_III_ hybridization in otherwise apomictic species, such as *Brachiaria decumbens*, *Panicum maximum*, *Poa pratensis*, *E. curvula*, *C. ciliaris*, *Pennisetum orientale* etc. (Bashaw and Hignight, 1990; Nogler, 1994; Naumova et al., 1999; Matzk et al., 2005; Kaushal et al., 2015a, 2018). In fact, B_III_ hybrids are formed directly, without intermediary of sexual relatives, and thus give rise to new apomictic biotypes, thereby further increasing the polymorphism of the agamic species complex (Nogler, 1994). Additionally, polyhaploids generated through the M_1_ pathway offer added advantage for understanding apomixis expression at diploid/haploid levels. Polyploid-polyhaploid cycles for generation and fixation of variability in natural polyploids have already been discussed (reviewed in Berthaud, 2001). Although rarely reported, partitioning also presents a possibility of obtaining sexual polyhaploids from apomictic polyploids where apomixis is under monogenic control, such as *P. maximum* (*Aaaa*). Such sexual polyhaploids would be a resource for breeding apomictic crops where naturally occurring apomicts are polyploids.

Recombination between apomixis components presents a system to study diversity in reproductive pathways of seed development. Such systems, when duly coupled with polyploidy, offer advantages for precise understanding of the various mechanisms, leading to interaction effects between apomixis components as well as their interface with genetic, epigenetic and environmental factors (Matzk et al., 2001, 2005; Aliyu et al., 2010; Hojsgaard, 2018). Additionally, it also serves as a stable system to generate newer cytotypes, through B_III_, M_1_ or HAPA (Kaushal et al., 2009, 2015a).

Although there are several reports whereby mutagenesis in natural apomictic plants converted them to sexual (Takahara et al., 2014, 2016), reports of a single mutation converting a sexual plant to apomictic are extremely rare (Chen et al., 2018; Gaafer et al., 2018). The strategy and application rely on generating (or inducing) individual components (say apomeiosis and parthenogenesis) separately and then attempting to reconstruct the apomixis phenotype by combining these components into one background. Organizing partitioned apomixis elements to develop an apomictic crop is a major challenge for plant breeders. In-fact, the most plausible approach to engineer apomixis into present day crop plants would be an applied synergy between “*evaluation*” and “*synthesis*” approaches. *Evaluation* precisely generates information from natural apomicts for identification of genes (apomixis *per se* or its components), gene actions and other required factors (e.g., environment, ploidy etc.), which may be appropriately utilized in “*synthesis*” approach to transfer/induce into sexually reproducing crops of interest, or to engineer key genes governing sexuality (Bicknell and Koltunow, 2004; Kaushal et al., 2004, 2005; Barcaccia and Albertini, 2013; Hand and Koltunow, 2014; Mieulet et al., 2016; Khanday et al., 2019). Identification of key master-regulatory sequences has been long sought, which may govern entry into apomeiotic/meiotic ES development as well as parthenogenetic/zygotic development of egg cell, however, the underlying mechanism is still poorly understood. Accordingly, based on recent discoveries of key genes, a strategy to introduce a transgene “apomixis cassette” containing dominant genes conferring to apomeiosis, parthenogenesis and autonomous development, has been proposed to generate an apomictic crop (Conner and Ozias-Akins, 2017). Two alternative pathways have been suggested: utilizing an artificial miRNA (amiRNA) *MiMe* cassette and an egg-specific promoter fused with a weak CENH3 variant cassette to generate a *MiMe* + GEM apomictic transgene line, or using amiRNA *MiMe* cassette to create a *MiMe* + *PsASGR-BBML* apomictic transgene line. MiMe lines may generate unreduced egg cells by replacing meiosis with mitosis (d’Erfurth et al., 2009), while CENH3/GEM (Ravi and Chan, 2010; Marimuthu et al., 2011) and *PsASGR-BBML* (Conner et al., 2015) may induce parthenogenesis. The endosperm in these cases will maintain the required 2*m*:1*p* ratio. Partial apomixis in rice has been recently achieved by triggering parthenogenesis in MiMe generated unreduced female gametes by ectopic expression of a male specific *OsBBM* gene in unfertilized ovules (Khanday et al., 2019). Another plausible approach to develop an apomictic cereal could be to introduce/reassemble apomeiosis and parthenogenesis, along with AED, however, to avoid gene flow, the final genotype must be male sterile (Kaushal et al., 2004).

## Conclusion

Apomictic mode of reproduction is seemingly a complex phenomenon, whereby the eventual expression depends on numerous major and minor factors, in addition to genotypic effects. Availability of recombination potential between its three components (apomeiosis, parthenogenesis and functional endosperm-development) offers advantages for understanding the origin, evolution, genetics and molecular biology of the phenomenon. With the increasing state of knowledge and efficient technological back-up, the biology of these components, independent as well as when linked, has been subjected to intense investigations. Large scale characterization of the reproductive diversity in agamic complexes is expected to unravel detailed insights into the possibility of partitioning. Amongst the components, apomeiosis has been investigated in detail, however, information on parthenogenesis and endosperm development is still inadequate. This is also important in a view to developing a universal model for generating apomictic crops. Comparison of molecular mechanisms governing apomeiosis, parthenogenesis and relaxed endosperm imprinting in apomicts, as compared to development of unreduced egg cell, haploid embryos and endosperm development in sexual crops through alternate pathways (e.g., restitution nuclei, endomitosis, uniparental chromosome-elimination, alloplasmic systems), is expected to yield important insights into possible overlaps during the seed formation process. Identification of the master regulatory switch triggering apomixis in sexual crops and sexuality in apomictic crops is a plant breeders’ dream. Though amalgamation of information gathered from apomictic and sexual systems (evaluation and synthesis approach) has led to the proposed model towards developing apomictic crops (Pupilli and Barcaccia, 2012; Conner and Ozias-Akins, 2017; Khanday et al., 2019), signaling pathways, cell-to-cell interactions (Juranic et al., 2018), and protein and metabolome investigations may greatly strengthen the state of knowledge.

## Author Contributions

PK involved in conceptualization, literature collection, compilation and writing the manuscript. KD, AR, MS, and VK performed literature collection and wrote the manuscript. AR and DM performed compilation and wrote the manuscript.

## Conflict of Interest Statement

The authors declare that the research was conducted in the absence of any commercial or financial relationships that could be construed as a potential conflict of interest.
